# Inferior vena cava thrombosis during extracorporeal membrane oxygenation: a case report and review of the literature

**DOI:** 10.1186/s13256-021-03057-0

**Published:** 2021-10-18

**Authors:** YouLian Chen, HuaiSheng Chen, XueYan Liu, ChengYing Hong, HuaDong Zhang

**Affiliations:** Present Address: Department of Critical Care Medicine, Shenzhen People’s Hospital, The Second Clinical Medical College of Jinan University, The First Affiliated Hospital of South University of Science and Technology, No.1017, Dong Men North Road, Luohu District, Shenzhen, 518020 China

**Keywords:** Extracorporeal membrane oxygenation, Inferior vena cava thrombosis, Acute respiratory distress syndrome, Cardiogenic shock

## Abstract

**Background:**

Extracorporeal membrane oxygenation (ECMO) is an effective cardiopulmonary support therapy, which can provide temporary cardiopulmonary support for critically ill patients whose condition cannot be reversed by conventional therapy. However, there are many complications in the use of ECMO, such as bleeding, thrombosis, and so on. Among them, inferior vena cava (IVC) thrombosis which can cause pulmonary embolism is a rare complication, which may be life-threatening.

**Case presentation:**

A 75-year-old female patient (Han Chinese ethnicity) with acute heart failure due to acute myocardial infarction in our department was retrospectively analyzed. After regular treatment was unsuccessful, she was treated with venoarterial ECMO (VA-ECMO). After her condition improved, she was withdrawn from ECMO and experienced a complication of IVC thrombosis. Enoxaparin was given immediately for 1 mg/kg every 12 hours hypodermic injection. The thrombus disappeared after anticoagulant therapy. She was discharged on the 60th day. Her level of consciousness returned to normal without residual central nervous system-related complications.

**Conclusions:**

IVC thrombosis is one of the possible serious complications in the process of ECMO therapy. Prevention of thrombosis and optimization of the anticoagulant regimen are the main preventive measures. Anticoagulant therapy is still the main treatment of IVC thrombosis in the process of ECMO therapy. Other interventional strategies need to accumulate clinical experience.

## Background

Extracorporeal membrane oxygenation (ECMO) is an effective cardiopulmonary support therapy, which can provide temporary cardiopulmonary support for critically ill patients, help improve hypoxia, buy more time for the treatment of primary diseases, and save many dying patients whose condition cannot be reversed by conventional therapy [1].

However, there are many complications associated with the use of ECMO, such as acute kidney injury, bleeding, thrombosis, and so on [2]. Inferior vena cava (IVC) thrombosis is a rare complication, but pulmonary embolism caused by thrombus shedding may be life-threatening. There are few reports and no standardized consensus on prevention and treatment. This paper reports the diagnosis and treatment of a patient with IVC thrombosis during ECMO, and discusses the prevention and treatment of IVC thrombosis during ECMO through a literature review. Informed consent was obtained for all special treatment protocols involved.

## Case presentation

**Fig. 1:**
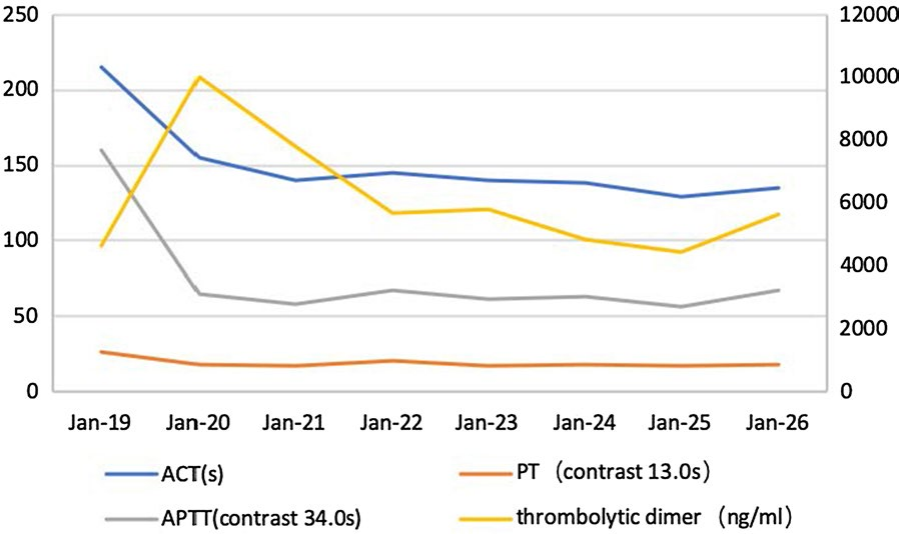
Monitoring values of anticoagulant dosage and coagulation function during extracorporeal membrane oxygenation

**Fig. 2:**
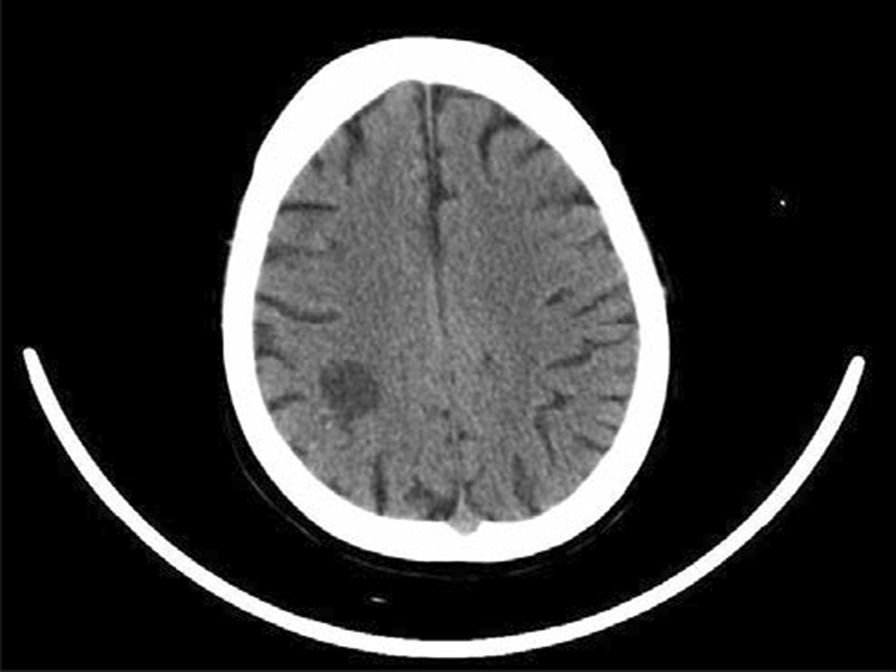
In the right parietal lobe, cerebral infarction and bleeding after a small amount of cerebral infarction may be possible

**Fig. 3:**
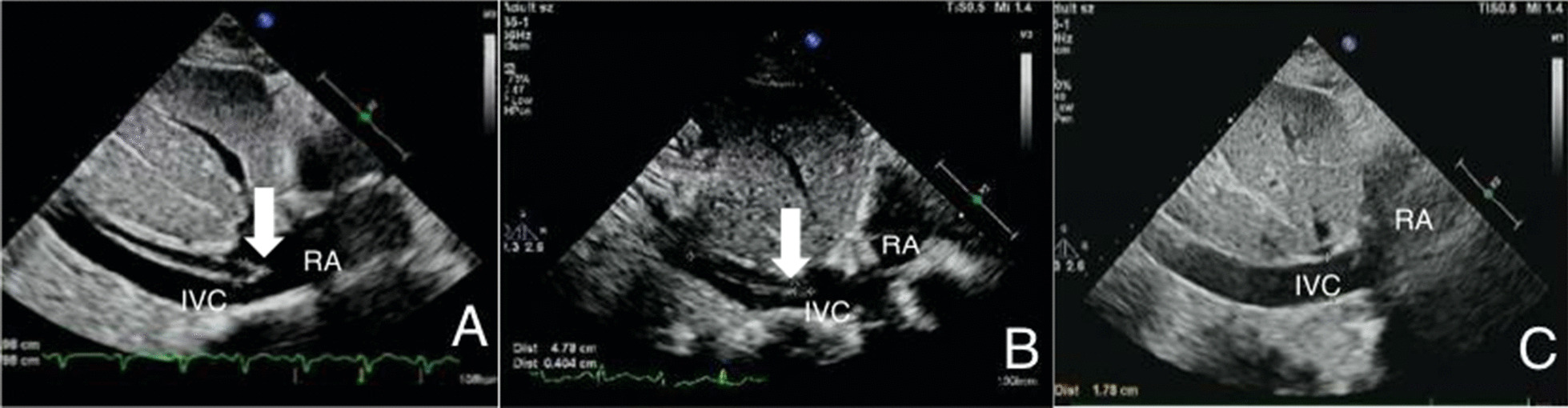
Echocardiography after cannula removal.** a** shows that after extracorporeal membrane oxygenation catheter removal, the inferior vena cava thrombosis was found by ultrasonography.** b** shows that the thrombosis of the inferior vena cava decreases gradually after heparin anticoagulation.** c** shows that the thrombus of inferior vena cava disappeared after heparin anticoagulation. *RA* right atrium, *IVC* inferior vena cava, Thrombosis in   inferior vena cava (arrowhead)

A 75-year-old female patient (Han Chinese ethnicity) weighing 55 kg with cardiogenic shock was admitted to our critical care department. After regular treatment was unsuccessful, she was treated with venoarterial extracorporeal membrane oxygenation (VA-ECMO) on January 19, 2021, for acute myocardial infarction complicated by cardiogenic shock. MAQUET ECMO equipment was used. The ECMO catheter was implanted into the right femoral vein and left femoral artery under ultrasound guidance. The end of the catheter was confirmed to the level of the right atrium by ultrasound. VA-ECMO was established with a rotational speed of 3500/minute, flow rate of 3.2 L/minute, fraction of inspired oxygen [FiO_2_] of 100%, gas flow rate of 4 L/minute, and vasoactive drugs dobutamine 6 μg/(kg/minute) and norepinephrine 0.1 μg/(kg/minute) The parameters of the ventilator were adjusted as follows: oxygen concentration 50%, respiratory rate 10 times/minute, tidal volume 5 mL/kg, and positive end-expiratory pressure 8 cmH_2_O. When ECMO was established, due to abnormal coagulation function (activated clotting time [ACT] > 200 seconds, activated partial thromboplastin time [APTT] > 160 seconds), anticoagulation was temporarily delayed. A total of 600 mL of plasma and 4 U of red blood cell suspension were supplemented. ACT was monitored every 2 hours. When ACT decreased to less than 200 seconds, heparin anticoagulation was started at APTT of 60 seconds (Fig. 1). We conducted a physical examination at the bedside, and we found that the pupil size of the patient’s eyes were not equal, so we performed a computed tomography (CT) examination. The CT report showed cerebral infarction and possibly a small amount of bleeding after infarction in the right parietal lobe. The right frontal lobe showed basal ganglia cavity infarction and elderly brain changes (Fig. 2). Because of the risk of intracerebral hemorrhage, we used a low-intensity heparin anticoagulation strategy. With the assistance of ECMO, the patient underwent coronary angiography + percutaneous coronary angioplasty + stent implantation aided by digital subtraction angiography (DSA) on January 20 at 08:20–09:35. After the operation, the cardiac function gradually recovered and the hemodynamics improved. On January 21, dopamine was stopped, and low-dose norepinephrine combined with dobutamine was continued. Because of the progressive decrease in platelets and the low activity of antithrombin III, the anticoagulation efficiency of unfractionated heparin was considered to decrease, and heparin-induced thrombocytopenia was not excluded. Argatroban anticoagulation was used to maintain APTT of between 50 and 70 seconds. On January 26, ECMO parameters were adjusted to a rotation speed of 2795 r/minute, blood flow 1.61 L/minute, FiO_2_% 21%, air flow 1.5 L/minute, and mean arterial pressure (MAP) 90 mmHg. Bedside echocardiography showed that the patient's cardiac systolic function was improved, left ventricular ejection fraction (LVEF) (biplane Simpson method) was 35%, and hemodynamics were stable. As the patient's cardiac function improved, we discussed with caregivers and family about the discontinuation of ECMO therapy, and the ECMO was removed after evaluation. On January 27, 2021, color Doppler ultrasound showed 34% LVEF (biplane Simpson method) and thrombosis from the IVC to the atrium (about 70 mm in length, 8 mm in width, with a floating tail end, Fig. 3a). Enoxaparin was given immediately for 1 mg/kg every 12 hours ih. On February 5, 2021, color Doppler echocardiography showed thrombosis from the IVC to the right atrium entrance (compared with January 27, 2021, volume reduced, shape changed, length about 48 mm, width about 4 mm, tail end floating, Fig. 3b). On February 19, 2021, color Doppler echocardiography showed no obvious abnormal echo in the IVC; combined with medical history, thrombus disappeared after anticoagulant therapy (Fig. 3c). Later, the patient's condition gradually stabilized. On the 30th day, she was taken off the ventilator and administered oral rivaroxaban instead of anticoagulants. Due to decreased muscle strength of her limbs, she received rehabilitation exercise and was discharged on the 60th day. Her level of consciousness returned to normal without residual central nervous system-related complications.

## Discussion

Although ECMO may be the most effective treatment for patients with severe heart or respiratory failure, it has many complications, including bleeding (33%), hemolysis (18%), and venous microthrombosis (10%) [3]. During ECMO, there are many risk factors for thrombosis, including disease severity, sedation, frequent blood transfusion, non-pulse blood flow, and blood exposure to a non-biological ECMO pipeline, all of which are the microenvironment leading to thrombosis, so anticoagulation therapy must be considered [4]. However, the incidence of bleeding during ECMO can be as high as 29%, with 10% at risk of massive bleeding and 4–10% at risk of intracranial hemorrhage. This group of patients may have insufficient anticoagulation due to the existence of bleeding risk factors, which is also an important reason for thrombosis [5]. Because of the possible risk of cerebral hemorrhage in our case, we reduced the anticoagulation intensity of heparin, which eventually led to IVC thrombosis.

In the process of ECMO treatment, we often encounter distal limb thrombosis. The treatment measures include strengthening anticoagulation, replacing the catheter, removing the thrombus, and other treatment measures, which are relatively easy to handle and of relatively low risk [6]. However, the incidence of thrombus formation near the entrance of the IVC is low, and thrombus shedding leads to acute massive pulmonary embolism, and even sudden death, which is rarely reported.

We retrospectively analyzed patients with IVC thrombosis during ECMO therapy reported in the previous literature. The PubMed database was searched by using the keywords "inferior vena cava thrombosis" and "extracorporeal membrane oxygenation." Our search showed 13 other reports on the subject (Table 1) [7–18]. Among the 14 patients, five patients were treated with VA-ECMO, mainly cardiogenic shock patients, including one heart transplant patient; the other eight patients were treated with veno-venous ECMO (VV-ECMO) after acute respiratory failure, including two lung transplant patients. Unfortunately, these two patients eventually died. The cause of death was not caused by pulmonary embolism, which may be related to the severity of the disease. According to the literature, the causes of IVC thrombosis are as follows: blood loss, insufficient volume, decreased blood flow velocity [14], continuous catheter shaking, low-intensity anticoagulation, or no heparin anticoagulation, all of which are also important reasons for thrombosis on the surface of ECMO catheter. In addition, because there is no heparin coating on the outer surface of the ECMO catheter, thrombosis is more likely to occur. If the antithrombin III (ATIII) level is low, there is also a risk of thrombosis; when the ATIII < 60% (normal range 80–120%). The anticoagulant efficiency of heparin decreased, and conventional ACT or APTT could not accurately determine the anticoagulant intensity, leading to thrombosis [10].

**Table 1:** Case reports of inferior vena cava thrombosis during extracorporeal membrane oxygenation

Year	Author	Thrombus site	Cause	Modality of ECMO	Intervention	Complication	Outcome
1997	Riccabona *et al*. [7]	IVC	Heart failure	VA-ECMO	Anticoagulation	No	Survival
2002	Jack *et al*. [8]	IVC	Lung transplantation	VV-ECMO	None	No	Death
2006	Bruno Mégarbane *et al*. [9]	IVC	Cardiogenic shock	VA-ECMO	Anticoagulation	No	Survival
2010	Alicia Sievert *et al*. [10]	IVC	ARDS	VV-ECMO	Anticoagulation	No	Survival
2011	Sylvain Beurtheret *et al*. [11]	IVC	Respiratory failure	VV-ECMO	Anticoagulation	PE	Survival
2015	Philippe Morimont *et al*. [12]	RA	Heart transplantation	VA-ECMO	Operation	No	Survival
2015	Thomas Bein *et al*. [13]	IVC	ARDS	VV-ECMO	Anticoagulation	No	Survival
2017	Cristina Ruisanchez *et al*. [14]	IVC	ARDS	VV-ECMO	Anticoagulation	No	Survival
2017	Samantha Wills *et al*. [15]	IVC	ARDS	VV-ECMO	Anticoagulation	No	Survival
2018	Magdy *et al*. [16]	IVC	Respiratory failure	VV-ECMO	Operation	No	Survival
2020	Marin Pavlov *et al*. [17]	IVC	Cardiogenic shock	VA-ECMO	Anticoagulation	No	Survival
2021	Ting Chen *et al*. [18]	IVC	Lung transplantation	VV-ECMO	Anticoagulation	No	Death

*ECMO* extracorporeal membrane oxygenation, *VA* venoarterial, *VV* veno-venous, *ARDS* acute respiratory distress syndrome, *IVC* inferior vena cava, *PE* pulmonary embolism, *RA* right atrium

The clinical manifestations of IVC thrombosis in ECMO patients were nonspecific, mainly manifested as "IVC syndrome," and distal limb edema in some patients after removal of the ECMO catheter [13]. In addition, if IVC thrombosis occurs during ECMO, sudden hypoxemia and ECMO flow decrease may also be caused by the patient's postural changes [14]. Hemolysis has also been reported [15].

The main diagnostic methods were ultrasound examination of the heart and IVC [7, 16]. It is worth noting that there is a kind of thrombus formed outside the ECMO catheter, which is not easy to find during ECMO, because it can be easily mistaken for the ECMO catheter wall. After the ECMO catheter was removed, two-dimensional ultrasound showed that the thrombus became tubular; color Doppler ultrasound showed that the blood flow could pass through the thrombus, and even the tail of the thrombus could float, as shown in the ultrasound in our case (Fig. 3a) [17, 18]. At present, there is no good method to identify this kind of thrombus, which may be judged by repeatedly measuring the wall thickness of the ECMO catheter. Therefore, color Doppler ultrasound can provide a suitable method for the detection and follow-up of venous thrombosis during ECMO or after removal of the ECMO catheter to prevent the occurrence of serious sequelae, such as pulmonary embolism.

Treatment measures include drug therapy, vascular interventional therapy, and surgery. According to a literature analysis, 76.9% (10/13) of patients received anticoagulant therapy, and there is no report about thrombolysis at present. One patient undergoing thrombectomy was a heart transplant patient, and another had received lung transplantation, and their prognosis was good. Both of the deaths were lung transplant patients [8, 18]. This indicates that the severity of the disease is an important factor affecting patient survival. At present, there is no report on the application of vascular interventional therapy. However, it has been reported that the AngioVac System can successfully evacuate the thrombus in cases of ECMO-related thrombosis [19].

## Conclusions

Based on our clinical cases and literature analysis, we believe that IVC thrombosis is one of the serious complications associated with the process of ECMO therapy. Once it occurs, it is closely related to the mortality and disability rate of patients. Prevention of thrombosis and optimization of the anticoagulant regimen are the main preventive measures, and anticoagulant therapy is still the main cause of IVC thrombosis in the process of ECMO therapy. Other interventional strategies need to accumulate clinical experience.

## Data Availability

Not applicable.
